# GO‐FAANG meeting: a Gathering On Functional Annotation of Animal Genomes

**DOI:** 10.1111/age.12466

**Published:** 2016-07-24

**Authors:** Christopher K. Tuggle, Elisabetta Giuffra, Stephen N. White, Laura Clarke, Huaijun Zhou, Pablo J. Ross, Hervé Acloque, James M. Reecy, Alan Archibald, Rebecca R. Bellone, Michèle Boichard, Amanda Chamberlain, Hans Cheng, Richard P.M.A. Crooijmans, Mary E. Delany, Carrie J. Finno, Martien A. M. Groenen, Ben Hayes, Joan K. Lunney, Jessica L. Petersen, Graham S. Plastow, Carl J. Schmidt, Jiuzhou Song, Mick Watson

**Affiliations:** ^1^ Department of Animal ScienceIowa State University806 Stange RoadAmesIA50011USA; ^2^ GABIINRAAgroParisTechUniversité Paris‐Saclay78350Jouy‐en‐JosasFrance; ^3^ USDA‐ARS Animal Disease Research UnitPullmanWA99164USA; ^4^ Department of Veterinary Microbiology & PathologyWashington State UniversityPullmanWA99164USA; ^5^ Center for Reproductive BiologyWashington State UniversityPullmanWA99164USA; ^6^ European Molecular Biology LaboratoryEuropean Bioinformatics InstituteWellcome Trust Genome CampusHinxtonCambridgeCB10 1SDUK; ^7^ Department of Animal ScienceUniversity of CaliforniaDavisCA95616USA; ^8^ INRAUMR1388 GénétiquePhysiologie et Systèmes d'ElevageF‐31326Castanet TolosanFrance; ^9^ The Roslin Institute and Royal(Dick) School of Veterinary StudiesUniversity of EdinburghEaster BushEdinburghEH29 9RGUK; ^10^ Department of Population Health and ReproductionVeterinary Genetics LaboratorySchool of Veterinary MedicineUniversity of California‐DavisDavisCAUSA; ^11^ Department of Economic Development, Jobs, Transport and ResourcesAgribiosciences BuildingBundooraAustralia; ^12^ Avian Disease and Oncology LaboratoryUSDAARSEast LansingMI48823USA; ^13^ Animal Breeding and Genomics CentreWageningen UniversityPO Box 3386700AH WageningenThe Netherlands; ^14^ Department of Population Health and ReproductionUniversity of CaliforniaDavisCA95616USA; ^15^ Queensland Alliance for Agriculture and Food InnovationCentre for Animal ScienceThe University of QueenslandSt. Lucia, 4072QueenslandAustralia; ^16^ Animal Parasitic Diseases LaboratoryBARCARSUSDABeltsvilleMD20705USA; ^17^ Department of Agricultural, Food, and Nutritional ScienceUniversity of AlbertaEdmontonABCanada; ^18^ Department of Agricultural, Food, and Nutritional ScienceUniversity of AlbertaEdmontonABCanada; ^19^ Department of Animal and Food SciencesUniversity of DelawareNewarkDE19716USA; ^20^ Department of Animal and Avian SciencesUniversity of MarylandCollege ParkMD20742USA

**Keywords:** data coordination centre (DCC), data sharing, Genomics, metanalysis

## Abstract

The Functional Annotation of Animal Genomes (FAANG) Consortium recently held a Gathering On FAANG (GO‐FAANG) Workshop in Washington, DC on October 7–8, 2015. This consortium is a grass‐roots organization formed to advance the annotation of newly assembled genomes of domesticated and non‐model organisms (www.faang.org). The workshop gathered together from around the world a group of 100+ genome scientists, administrators, representatives of funding agencies and commodity groups to discuss the latest advancements of the consortium, new perspectives, next steps and implementation plans. The workshop was streamed live and recorded, and all talks, along with speaker slide presentations, are available at www.faang.org. In this report, we describe the major activities and outcomes of this meeting. We also provide updates on ongoing efforts to implement discussions and decisions taken at GO‐FAANG to guide future FAANG activities. In summary, reference datasets are being established under pilot projects; plans for tissue sets, morphological classification and methods of sample collection for different tissues were organized; and core assays and data and meta‐data analysis standards were established.

## What is FAANG?

Modern biology is transitioning from a descriptive to a predictive science, as we begin to learn the rules for outcomes (phenotypes) from specific genomic starting points (genotypes) (Ritchie *et al*. 2015). Over the past 15 years, there have been substantial cross‐agency and transnational investments in the sequencing of domesticated animal genomes; the challenge now is to understand the instructions encoded in such genomes and to predict the resulting phenotypes. Such signals consist of specific sequences as well as epigenetic modifications to DNA and chromatin. Given the massive number of quantitative phenotypes that can arise from the interaction between environment and genomes, this ‘genome‐to‐phenome challenge’ is a daunting task. Fortunately, new resources in genome sequence data and bioinformatic tools indicate that modeling phenotype from genotype is within our grasp. For example, genome‐wide association studies in human genetics report sequence variants associated with disease or quantitative traits are found mostly outside of coding regions of genes (Hindorff *et al*. 2009). The detailed annotation data from the ENCODE project has shown that such phenotype‐associated variants are often coincident with regulatory elements; intriguingly, chromatin conformation or transcription factor occupancy at these elements can often be tied directly to the specific cell or tissue biology under study (Maurano *et al*. 2012; Vernot *et al*. 2012; Kundaje *et al*. 2015). Such functional annotation at every region of the genome not only can help interpret any particular associated SNP but can also filter lists of associated SNPs that are difficult to rank through genetic means; i.e. SNPs in high linkage disequilibrium (Khurana *et al*. 2013).

However, biochemical decoding of function is most powerfully performed in parallel on the same samples and is an enormous undertaking requiring the concerted efforts of hundreds of scientists across the globe. To address this challenge, the FAANG Consortium (www.faang.org) developed from the grass roots and set out its goals in a recent white paper (Andersson *et al*. 2015). In this publication, the FAANG community provided a vision for a first phase of FAANG, including initial guidance on parallel deep sample and metadata collection from species with high‐quality genome assemblies as well as definition of specific data types and infrastructure needed for this initial research. In the 18 months before the GO‐FAANG Workshop, the critical organizational and communications infrastructure necessary for accomplishing the first phase of FAANG was built, with over 300 members joining the consortium worldwide. Importantly, membership in the following groups is open to all scientists interested in participating in FAANG and agreeing to established policies for data use (visit www.faang.org for details).

A steering committee was created to develop and implement strategies to advance the FAANG project, involving representatives from many countries, multiple continents, and species of research interest. Four specific activity committees were formed, including Animals, Samples and Assays (ASA); Metadata and Data Sharing (M&DS); Bioinformatics and Data Analysis (B&DA); and Communications (COM). These committees convene frequently by conference call to develop policies and standardize procedures to advance FAANG. Although excellent progress has been and continues to be made in these meetings, the GO‐FAANG Workshop was organized in recognition of the need to involve the wider community in these discussions to broaden and strengthen the benefits of consortium activities.

## The GO‐FAANG Workshop

The GO‐FAANG Workshop objectives included establishing priorities for research efforts; planning the management structures required for efficient use and sharing of samples, data and computational tools; and identifying resources needed to accomplish these goals. Three plenary talks and presentations by funding agency representatives set the stage for what is possible in functional annotation of genomes. An emphasis was then placed on small group discussions; these were designed to develop policies and approaches to maximize the success of this crucial next phase in animal genomics.

### Plenary talks set the stage

The workshop included plenary lectures by leaders in the genomics field (John Stamatoyannopoulos, University of Washington; Christine Wells, University of Glasgow; and Paul Flicek, European Molecular Biology Laboratory, European Bioinformatics Institute, EMBL‐EBI), who described the state of the art in genome function analysis in the human and mouse species. A recurring theme was that the domesticated animal community is well‐positioned to exploit the knowledge gained in human and model organism projects through adapting technologies and data analysis pipelines and in comparative analyses. In addition, several FAANG members described current status and future plans of the FAANG community. Reports on pilot FAANG projects in the US and France included the collection and initial analysis of selected tissues as well as development of plans for bioinformatic pipelines to collect, share and analyze data produced in the FAANG project.

### Funding agencies participated at multiple levels

An important component of FAANG success is the identification and nurturing of opportunities to gain support for annotation activities. Thus the involvement of funding agencies from several countries was critical. Representatives of the National Science Foundation, the U.S. Department of Agriculture, the Canadian Genome Enterprise, the National Institutes of Health, the Research Councils of the United Kingdom, as well as the European Commission, presented their perspectives on FAANG goals and provided information on research opportunities for FAANG projects. Several representatives also suggested mechanisms to create new opportunities for research funding, including international consortia to organize the research enterprise. These presentations provided clear evidence that FAANG is of interest to these agencies, boding well for future funding opportunities in animal genome annotation.

### Small group discussions expanded community input

#### Data creation group

During the session there was a vigorous discussion on what constituted ‘core tissues’, ‘core assays’ and associated standardized protocols. The community is confronted with the complexity of integrating the efforts at several institutions and among multiple principal investigators engaged in generating large tissue collections for genotype‐to‐phenotype studies. The wide phylogenetic range of species and tissues necessarily imposes targeted optimizations in terms of specific morphological classification and method of sample collection. Requirements vary between tissues [e.g. soft and relatively homogeneous tissues (liver) vs. rich in fat and/or highly heterogeneous tissues (mammary gland or brain)]. Utilization of snap‐frozen specimens currently limits the application and resolution of some methods (e.g. DNase‐seq and ATAC‐seq). Furthermore, some tissues can only be collected in amounts sufficient for low‐input assays.

Since 2015, members of the ASA Committee have collaborated with the M&DS Committee to define minimum requirements and suitable ontologies for organizing sample collections in the EMBL‐EBI BioSamples database (Gostev *et al*. 2012) and refer to the B&DA Committee for defining metrics for data quality assessment (see below). Following GO‐FAANG, a strategy was proposed to capitalize on efforts from independent projects or expertise on different assays (see ASA Committee's information on www.faang.org). The expected outcome is reducing redundancy and enhancing synergy through facilitated sharing of common samples for performing complementary assays, using a limited number of easily collected tissues that are the most relevant for the genotype‐to‐phenotype research projects of FAANG groups (ftp://ftp.faang.ebi.ac.uk/ftp/protocols). Central to this effort is the registration of all sample/data collections in the BioSamples database.

An item emerging from the discussions was that the FAANG community will benefit from developing interactions with partners actively involved in ENCODE and other consortia to efficiently transfer cutting‐edge technologies. For example, new or more powerful assays continue to emerge, greatly extending the range of methods both for basic and translational research (e.g. Buenrostro *et al*. 2015; Jin *et al*. 2015). Importantly, the interaction of FAANG with other consortia will enhance the value of domesticated animals across different research fields, for example as alternative biomedical models (Andersson *et al*. 2015).

#### Data analysis group

The role of the B&DA group is to agree and define standard pipelines for the analysis of FAANG data, with the aim that datasets produced by different laboratories are comparable. The breakout session began by discussing bioinformatics pipelines and whether these should be tightly or loosely defined. The challenge of having very specific, tightly defined software standards is that some collaborators may not be able to meet those standards and some analyses may need to be repeated. However, the results from similar but not identical (loosely defined) pipelines are not comparable and introduce batch effects, which may be indistinguishable from genuine effects. The consensus was that FAANG should adopt tightly defined standards for software pipelines to include software names, versions and all parameters used. The discussion then moved to data standards and considered standards for both data type (e.g. stranded RNA‐Seq) and meta‐data. Specifying such standards is essential to support the reuse of the data, but there exists a tension between rigid standards, which ensure all datasets will be comparable, and more relaxed standards that allow existing data to be included in the FAANG collection and continue to benefit from the existing investment made by the funders and investigators to generate this data. A possible compromise was discussed: data (past, present and future) could be allowed to meet different specifications within the standards, allowing older data to be validated and integrated into the collection, with any differences clearly annotated. Only data that had met the same specification of the standard would then be compared, and this would avoid the introduction of batch effects. This compromise would allow researchers to include previous data generated before the FAANG project began. The discussion also highlighted how standard reference datasets will be critical for evaluating how FAANG pipelines change over time, through demonstration that new methods may improve previous results.

Reproducibility of the FAANG pipelines is also essential to ensure that the whole community can benefit from such evaluations. The breakout session discussed the possibility of using virtual machine images or containerization solutions such as Docker (https://www.docker.com/). The advantage of either, for providing FAANG analysis pipelines, is that sharing specific pipelines is much easier, ensuring that the same analysis is done with the same program and version at every institution. The M&DS group strongly encourages the use of ontologies to describe samples and experiments, but an appropriate balance needs to be struck so that groups working on new or unusual samples or assays can still describe their experiments. There are technological solutions to this problem, and FAANG needs to investigate and implement as appropriate, ensuring the highest quality metadata without overburdening the data collectors.

A third topic was how FAANG can use incentives to stimulate appropriate collaborations. One mechanism under development is a generally available list of projects and grant applications that would enable (i) formation of appropriate collaborations with existing work and (ii) identification of gaps in existing work that could be filled. A second potential mechanism would be sharing of personnel between research groups that could advance existing projects in a host lab while simultaneously providing training for personnel to use on return to home research groups. Two recently funded groups, the National Science Foundation ‘Animal Genome to Phenome Research Coordination Network’ (AG2P: www.ag2p.org) and the COST Action ‘Functional Annotation of Animal Genomes – European network’ (FAANG‐Europe: http://www.cost.eu/COST_Actions/ca/CA15112) will provide a mechanism to support such visits for FAANG participants. Another mechanism of incentivizing collaborations is to encourage and enable the publication of results, standards and pipelines.

## Beyond GO‐FAANG

### Implementation of community discussions

The discussions at the GO‐FAANG meeting clearly demonstrated that the FAANG datasets must be well described. The M&DS committee worked during 2015 to develop FAANG metadata standards; version 1 was released in early 2016 (http://www.faang.org/groups?name=metadata). These standards define what metadata should be collected for samples, experiment and analyses run by FAANG and how that metadata should be structured. FAANG recommends all sample collection groups pre‐register their samples in the EMBL‐EBI BioSamples database (Gostev *et al*. 2012; https://www.ebi.ac.uk/biosamples/). The BioSamples database allows FAANG to provide structure to their sample records and create sample records for donors, tissues and cell samples. It allows relationships to be recorded so that sampling across pedigrees can be clearly recorded for downstream use. Another important aspect of well‐structured metadata is the use of appropriate ontology terms, ensuring that everyone can have a specific understanding of the animal, tissue and cell type collected by a group or the experiment conducted. FAANG looks to use actively maintained ontologies and aligns its recommendations with other large efforts such as ENCODE. The current ontology recommendations include the Livestock Breed Ontology, the Vertebrate Trait Ontology, UBERON and the Cell Ontology.

### Current FAANG projects are advancing and new projects have begun

**Figure 1:**
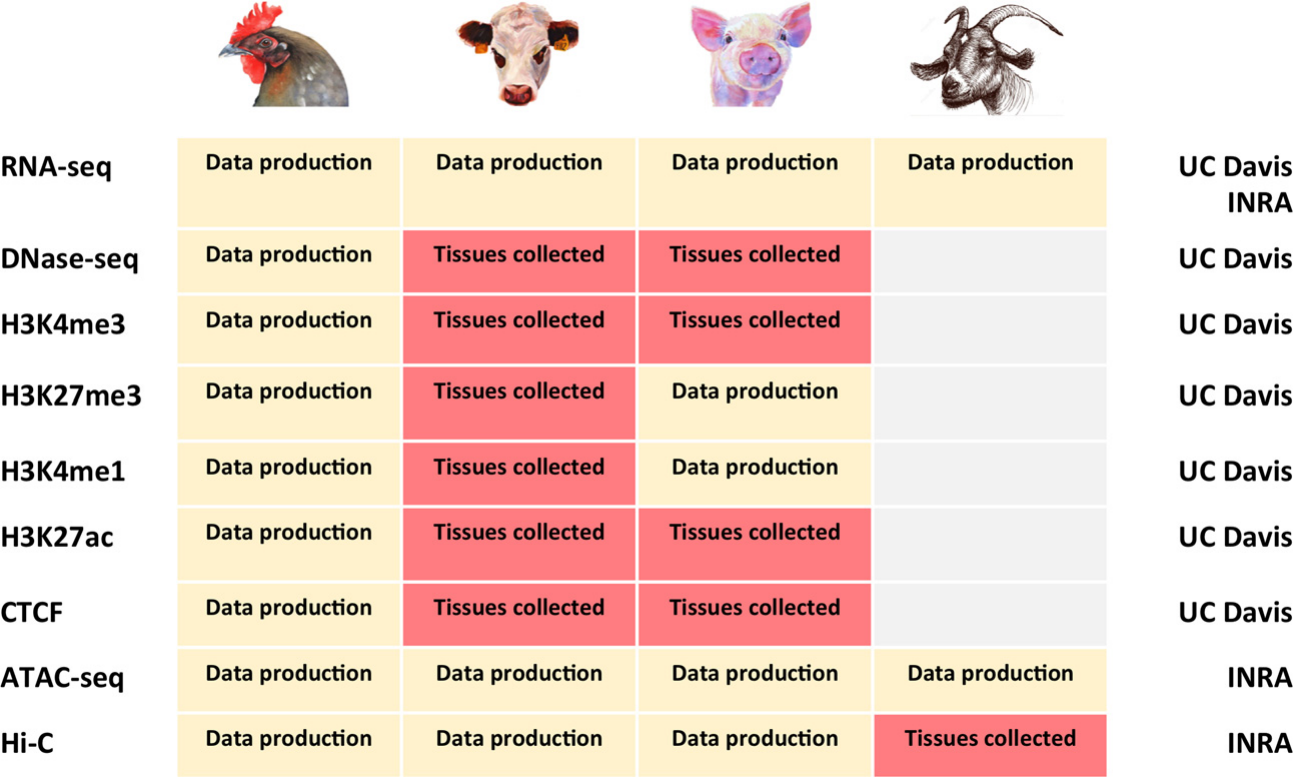
Summary of tissues collected and assays performed in two pilot FAANG research projects. See text for details.

Two FAANG pilot projects, coordinated by the Institut National de la Recherche Agronomique (INRA) in France and the University of California–Davis (UC Davis) in the USA, were started in 2015. These projects aim at identifying regulatory elements within domesticated animal genomes by refined functional annotation of biologically important representative tissues. For both projects, the first phase consisted of sampling of a wide variety of tissues from reference animals in four species: chicken, pig and cattle (both UC Davis and INRA) and goats (INRA only) (Fig. 1). Different protocols were used to perform multiple assays (histology, chromatin assays, gene expression, DNA conformation and accessibility). Specific representative tissues were selected [cerebellum, cortex, hypothalamus, liver, lung, adipose tissue, muscle and spleen for UC Davis; liver and immune cells (CD4+ and CD8+) for INRA] for core molecular assays development and data production (beginning in 2016). In addition to RNA‐seq, UC Davis focused on ChIP‐seq (histone marks and CTCF) and chromatin accessibility assays (DNase‐seq), whereas INRA focused on small RNA‐seq sequencing, chromatin accessibility assays (ATAC‐seq; Buenrostro *et al*. 2013), and genome‐wide Chromosome Conformation Capture (Hi‐C; Rao *et al*. 2014) (Fig. 1). Preliminary results for ChIP‐seq, ATAC‐seq and Hi‐C assay optimization and analysis were presented at the Plant and Animal Genome XXIV Conference during the FAANG Workshop (see http://faang.org/bbsdb/PAG2016/ASA_committee_Report.pdf).

Since 2015, new projects have joined the FAANG community (see http://faang.org/bbsdb/PAG2016/Eg_FAANG_associ_proj_2016.pdf). Most of these projects aim to identify the impact of genetic variation on the functional features of animal genomes, with the ultimate goal to identify causative variations affecting complex traits. In one, the ‘WUR‐pigENCODE’ project, led by Wageningen University (NL) in collaboration with the University of Illinois (US), is adding new functional annotations to previous methylome studies (Schachtschneider *et al*. 2015). In another, a complete range of FAANG annotation is being generated in parallel to eQTL and allele specific expression approaches in dairy cattle (Chamberlain *et al*. 2015). Two projects recently funded by the Genome Canada Competition – ‘Genomics and Feeding the Future’ – are linked to FAANG. These projects focus on feed efficiency in dairy cattle and disease resilience in pigs. The initial activities focus on the collection of FAANG‐ready samples (http://www.genomecanada.ca/sites/genomecanada/themes/genomecanada/backgrounders/BK-2014-competition.pdf). As well, members of the horse genome community were funded to start the development of a database of tissues to investigate gene expression and regulation in the healthy adult horse (http://www.grayson-jockeyclub.org/resources/newprojects.pdf). Finally, in response to FAANG member requests, a new webpage has been developed to provide information on new funding opportunities for FAANG‐related proposals (available on FAANG website, member's area page).

### Forthcoming FAANG meetings

A full‐day FAANG Symposium has been organized for July 23, 2016, which can be attended by registrants of either the Joint Annual Meeting of Animal Science Societies (https://asas.org/meetings/jam-2016/home) or the International Society of Animal Genetics (ISAG; https://asas.org/meetings/isag2016/home). A second FAANG Workshop will be held at the Plant and Animal Genome meeting in San Diego in 2017, and it is anticipated that a FAANG Workshop will be organized for the Dublin ISAG meeting in 2017. Details as they become available will be posted on the FAANG website.

## Future goals

As biology further transitions from a descriptive to a predictive science, collection and interpretation of genomic data across a wider range of species is contributing significantly. Although datasets obtained from easily collected tissues are a start on this long journey, datasets from additional tissues and assays are expected to come on‐line as future funding becomes available. Additionally, groups are beginning to implement extensive antemortem and postmortem phenotyping of animals and tissues, including histologic assessment of tissue sections to be used for assays, e.g. to accurately account for the absence of subclinical disease while defining cell populations. This will represent an important step forward in the second phase of FAANG (i.e. studying how environmental, physiological and genetic variation affects regulatory elements; Andersson *et al*. 2015). Thus FAANG's data will drive forward predictive biology, particularly in the genomic selection arena for the domesticated species.

Further, the resources developed here lower the activation threshold for emerging model organism communities to initiate genome annotation. FAANG should therefore increase understanding of the basic biological principles behind functional conservation and divergence across the different species involved. Such integrative analysis of this data will also result in new analysis techniques as well as updated standards for data re‐use. Taken together, the insights gained from this initiative will have positive impacts within biological/genetic research, which will help researchers address the genotype‐to‐phenotype challenge.

## References

[age12466-bib-0001] Andersson L. , Archibald A.L. , Bottema C.D. *et al* (2015) Coordinated international action to accelerate genome‐to‐phenome with FAANG, the Functional Annotation of ANimal Genomes project. Genome Biology 16, 57. 25854118 10.1186/s13059-015-0622-4 PMC4373242

[age12466-bib-0002] Buenrostro J.D. , Giresi P.G. , Zaba L.C. , Chang H.Y. & Greenleaf W.J. (2013) Transposition of native chromatin for fast and sensitive epigenomic profiling of open chromatin, DNA‐binding proteins and nucleosome position. Nature Methods 10, 1213–8. 24097267 10.1038/nmeth.2688 PMC3959825

[age12466-bib-0003] Buenrostro J.D. , Wu B. , Litzenburger U.M. , Ruff D. , Gonzales M.L. , Snyder M.P. , Chang H.Y. & Greenleaf W.J. (2015) Single‐cell chromatin accessibility reveals principles of regulatory variation. Nature 523, 486–90. 26083756 10.1038/nature14590 PMC4685948

[age12466-bib-0004] Chamberlain A.J. , Vander Jagt C.J. , Hayes B.J. , Khansefid M. , Marett L.C. , Millen C.A. , Nguyen T.T. & Goddard M.E. (2015) Extensive variation between tissues in allele specific expression in an outbred mammal. BMC Genomics 16, 993. 26596891 10.1186/s12864-015-2174-0 PMC4657355

[age12466-bib-0005] Gostev M. , Faulconbridge A. , Brandizi M. , Fernandez‐Banet J. , Sarkans U. , Brazma A. & Parkinson H. (2012) The BioSample Database (BioSD) at the European Bioinformatics Institute. Nucleic Acids Research 40, D64–70. 22096232 10.1093/nar/gkr937 PMC3245134

[age12466-bib-0006] Hindorff L.A. , Sethupathy P. , Junkins H.A. , Ramos E.M. , Mehta J.P. , Collins F.S. & Manolio T.A. (2009) Potential etiologic and functional implications of genome‐wide association loci for human diseases and traits. Proceedings of the National Academy of Sciences of the United States of America 106, 9362–7. 19474294 10.1073/pnas.0903103106 PMC2687147

[age12466-bib-0007] Jin W. , Tang Q. , Wan M. *et al* (2015) Genome‐wide detection of DNase I hypersensitive sites in single cells and FFPE tissue samples. Nature 528, 142–6. 26605532 10.1038/nature15740 PMC4697938

[age12466-bib-0008] Khurana E. , Fu Y. , Colonna V. *et al* (2013) Integrative annotation of variants from 1092 humans: application to cancer genomics. Science 342, 1235587. 24092746 10.1126/science.1235587 PMC3947637

[age12466-bib-0009] Roadmap Epigenomics Consortium , Kundaje A. , Meuleman W. *et al* (2015) Integrative analysis of 111 reference human epigenomes. Nature 518, 317–30. 25693563 10.1038/nature14248 PMC4530010

[age12466-bib-0010] Maurano M.T. , Humbert R. , Rynes E. *et al* (2012) Systematic localization of common disease‐associated variation in regulatory DNA. Science 337, 1190–5. 22955828 10.1126/science.1222794 PMC3771521

[age12466-bib-0011] Rao S.S. , Huntley M.H. , Durand N.C. *et al* (2014) A 3D map of the human genome at kilobase resolution reveals principles of chromatin looping. Cell 159, 1665–80. 25497547 10.1016/j.cell.2014.11.021 PMC5635824

[age12466-bib-0012] Ritchie M.D. , Holzinger E.R. , Li R. , Pendergrass S.A. & Kim D . (2015) Methods of integrating data to uncover genotype‐phenotype interactions. Nature Reviews Genetics 16, 85–97. 10.1038/nrg3868 25582081

[age12466-bib-0013] Schachtschneider K.M. , Madsen O. , Park C. , Rund L.A. , Groenen M.A. & Schook L.B. (2015) Adult porcine genome‐wide DNA methylation patterns support pigs as a biomedical model. BMC Genomics 16, 743. 26438392 10.1186/s12864-015-1938-x PMC4594891

[age12466-bib-0014] Vernot B. , Stergachis A.B. , Maurano M.T. , Vierstra J. , Neph S. , Thurman R.E. , Stamatoyannopoulos J.A. & Akey J.M. (2012) Personal and population genomics of human regulatory variation. Genome Research 22, 1689–97. 22955981 10.1101/gr.134890.111 PMC3431486

